# A plethora of *Plasmodium* species in wild apes: a source of human infection?

**DOI:** 10.1016/j.pt.2011.01.006

**Published:** 2011-05

**Authors:** Julian C. Rayner, Weimin Liu, Martine Peeters, Paul M. Sharp, Beatrice H. Hahn

**Affiliations:** 1 Malaria Programme, Wellcome Trust Sanger Institute, Wellcome Trust Genome Campus, Hinxton, Cambridge, CB10 1SA, UK; 2 Department of Medicine, University of Alabama at Birmingham, Birmingham, Alabama 35294, USA; 3 Department of Microbiology, University of Alabama at Birmingham, Birmingham, Alabama 35294, USA; 4 Institut de Recherche pour le Développement (IRD) and University of Montpellier 1, 34394 Montpellier, France; 5 Institute of Evolutionary Biology and Centre for Immunity, Infection and Evolution, University of Edinburgh, Edinburgh EH9 3JT, UK

## Abstract

Recent studies of captive and wild-living apes in Africa have uncovered evidence of numerous new *Plasmodium* species, one of which was identified as the immediate precursor of human *Plasmodium falciparum*. These findings raise the question whether wild apes could be a recurrent source of *Plasmodium* infections in humans. This question is not new, but was the subject of intense investigation by researchers in the first half of the last century. Re-examination of their work in the context of recent molecular findings provides a new framework to understand the diversity of *Plasmodium* species and to assess the risk of future cross-species transmissions to humans in the context of proposed malaria eradication programs.

## Ape *Plasmodium* infections: a historical perspective

*Plasmodium* parasites are extraordinarily successful and infect many vertebrate hosts, ranging from reptiles to birds to mammals. Our closest relatives, the African great apes, are no exception. While working in Cameroon in 1917, Eduard Reichenow observed three morphologically distinct *Plasmodium* parasites in the blood of chimpanzees (*Pan troglodytes*) and gorillas (*Gorilla gorilla*) [1], a finding soon confirmed by Saul Adler and Donald Blacklock in Sierra Leone [2,3]. In the following decades, *Plasmodium* parasites in gorillas and chimpanzees were studied at several sites across west and central Africa [4–9]. The consensus reached from this work was that there were indeed three *Plasmodium* species infecting African great apes, and that these were distinguishable based on their life-cycle and morphology. One species bore a striking resemblance to human *P. falciparum*, so much so that it was initially assumed to be the same species; only later was it accepted to be distinct, and named *P. reichenowi* [9]. A second parasite in apes, *P. rhodaini,* closely resembled *P. malariae* found in humans, whereas the third, *P. schwetzi*, was more difficult to characterize and was referred to as *P. vivax-*like or *P. ovale*-like by different investigators [10,11]. Recognizing that *P. falciparum* and *P. reichenowi* were quite distinct from the other known *Plasmodium* species, it was suggested more than 50 years ago that the former should be placed in a separate genus, *Laverania* [12]. Although this taxonomy was not widely adopted, the term has recently been resurrected and is useful as a subgenus designation [13].

Surprisingly, genetic material was obtained for only one of these three *Plasmodium* species found in apes. In 1968, a chimpanzee that had been imported into the USA was found to be infected with *P. reichenowi*. The parasite was isolated at the Centers for Disease Control (CDC) and subsequently propagated for molecular and biological analyses [14]. This *P. reichenowi* isolate has been used for numerous comparative genetic studies [15–18], ranging from single-gene to whole-genome analyses, for example, to identify genes in *P. falciparum* that are under differential selection pressures [19]. A full-length genome sequence is now nearing completion (M. Berriman, personal communication). Although *P. schwetzi* was also isolated by the CDC [11,20], no aliquots of this isolate apparently remain, and no DNA was ever recovered from this specimen (J.W. Barnwell, personal communication). *P. rhodaini* was never genetically characterized.

## A molecular revolution: ape parasites revisited

This view of the three species of *Plasmodium* in apes from west and central Africa (*P. reichenowi*, *P. schwetzi* and *P. rhodaini*), which mirror the three human *Plasmodium* species (*P. falciparum*, *P. ovale* and *P. malariae*) found in the same regions, has been completely revised in the past two years. The first suspicion that the old descriptors might not be sufficient came from the identification of parasites morphologically resembling *P. falciparum* in two pet chimpanzees from Gabon [21]. When the complete mitochondrial genome of one of these parasites was sequenced, it became clear that it was related to, but quite divergent from, *P. falciparum* and the CDC isolate of *P. reichenowi*. This led to the proposal of a new species, *P. gaboni*. Four additional studies rapidly followed that also reported sequences from *P. falciparum*-like parasites in African apes [13,22–24]. All of these pointed to the existence of additional species within the *Laverania* subgenus [13,21–24], but their precise number and true host associations were unclear. For example, various chimpanzee-derived lineages were all placed in one species [22], or split into four species [13,24]. In addition, only relatively few samples were analyzed, most of which were derived from sanctuary apes that could have become infected in captivity. Finally, some of the new sequences were clearly polymerase chain reaction (PCR)-induced hybrids which formed lineages intermediate between clades [23].

**Figure 1:**
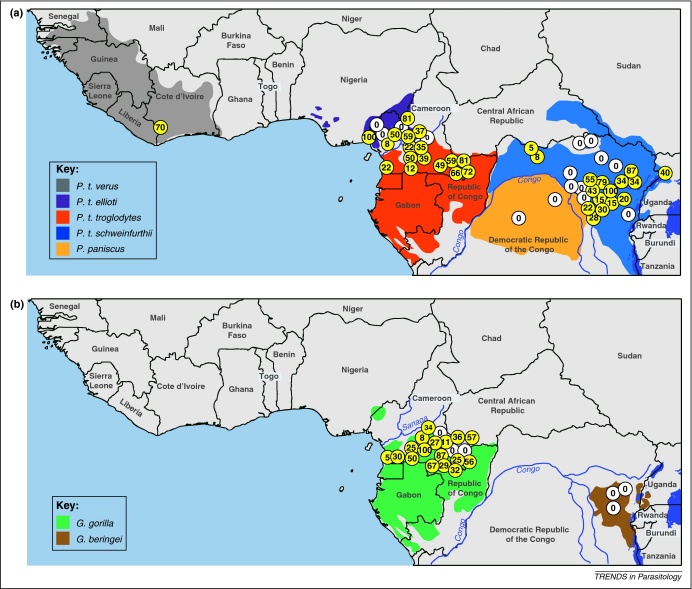
Geographic distribution, species association and prevalence of ape *Plasmodium* infections in sub-Saharan Africa. Field sites where wild-living chimpanzees and bonobos **(a)**, or gorillas **(b)** were sampled are shown. Sites where ape *Plasmodium* infections were detected are highlighted in yellow, with the estimated prevalence indicated [25,30]. The upper panel depicts the ranges of the four subspecies of the common chimpanzee (*Pan troglodytes verus*, gray; *P. t. ellioti,* magenta; *P. t. troglodytes*, red; and *P. t. schweinfurthii*, blue) and of the bonobo (*P. paniscus*, orange). The lower panel depicts the ranges of western (*Gorilla gorilla*, green) and eastern (*G. beringei*, brown) gorillas (map courtesy of Lilian Pintea, The Jane Goodall Institute, Arlington, Virginia, USA.).

**Figure 2:**
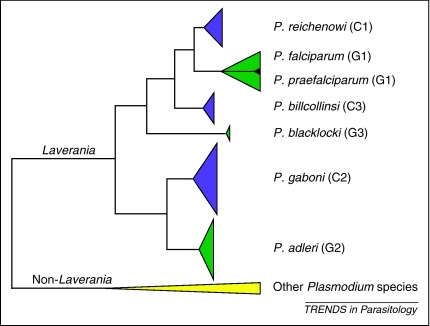
Phylogeny of ape-derived *Plasmodium* mitochondrial sequences: identifying six clades within the *Laverania* subgenus. A summary representation of an analysis of nearly 700 SGA-derived ape *Plasmodium cytb* sequences is shown (modified from Supplementary Figure 3 in [25]). Clades are summarized as triangles; the height of each triangle reflects the number of sequences, whereas the depth denotes the maximum divergence within each clade. Green and magenta colors highlight *Laverania* parasites from gorillas (G1–G3) and chimpanzees (C1–C3), respectively (non-*Laverania* parasites are shown in yellow). Each clade reflects a distinct species; pre-existing or proposed names for these species are shown on the right. The black triangle indicates human *P. falciparum* sequences, which form a monophyletic clade within the G1 radiation of gorilla parasites.

These uncertainties have since been resolved by a large-scale molecular epidemiological study [25] that used non-invasive methods (Box 1) to examine nearly 3,000 fecal samples from wild-living apes at multiple sites across Africa (Figure 1). PCR primers were designed to amplify *Plasmodium* mitochondrial, apicoplast and nuclear sequences from ape fecal DNA. Importantly, a single-genome amplification (SGA) approach was used to preclude *in vitro* artifacts that frequently occur when conventional PCR is used to amplify mixtures of genetically diverse templates (as is the case in many *Plasmodium* infections) [25]. The study revealed that wild apes harbored parasites that fell into six discrete major clades within the *Laverania* subgenus (Figure 2). The host specificity of these clades was intriguing: sequences from three of these lineages were found only in chimpanzee samples, whereas sequences from the other three clades were found only in samples from western gorillas (*G. gorilla*), even when both of these host species were sampled at the same geographical location. In contrast, *Plasmodium* sequences were not detected in eastern gorillas (*G. beringei*) or bonobos (*P. paniscus*) despite several hundred samples being tested [25]. The samples taken from eastern gorillas were collected at sites no more than 500–700 m above sea level. Hence, the absence of *Laverania* parasites was not simply a function of high altitude.

## A minimum of six ape *Laverania* species

Naming these various *Laverania* clades as different *Plasmodium* species on the basis of genetic information alone is likely to be controversial, but seems necessary at this time. The endangered status of African apes will make it difficult to obtain infected blood to derive *Plasmodium* isolates. Moreover, the high frequency of mixed-species infections (see below) will confound meaningful correlations of parasite morphology with genetic information from the same sample. Thus, it is unclear if and when the morphological and biological data traditionally used for the taxonomic description of new *Plasmodium* species will become available. However, there is a pressing need for a standardized nomenclature to provide a common framework for future research because there is confusion surrounding recently proposed species names. We believe that a strong case can be made that the six clades of ape *Plasmodium* sequences reflect distinct parasite species. Nevertheless, we recognize that the proposal described below will remain tentative until a full taxonomic evaluation has been completed.

Several lines of evidence indicate that the six lineages within *Laverania* each represent a distinct species. First, the various mtDNA clades are at least as divergent from each other as are *P. falciparum* and *P. reichenowi* [25], which in turn are much more divergent than the two recently proposed species within *P. ovale* [26]. Second, as noted above, the clades exhibit remarkably strict host specificity, indicating isolation at least between the parasites infecting chimpanzees and gorillas. Third, both of these aspects of the mtDNA phylogeny are recapitulated by sequences from the two other parasite genomes, i.e. the *clpC* gene from apicoplast DNA and the *ldh* gene from the nuclear genome, despite the fact that fewer *clp*C and *ldh* sequences were analyzed [25]. The apicoplast genome appears to be uniparentally inherited in the same fashion as mtDNA [27–29]. However, biparentally inherited nuclear genes can assort independently of the organelle genomes and so the *ldh* data in particular indicate that the division into six clades is not an artifact of the mode of mtDNA inheritance. Statistical support for the clades in the *clpC* and *ldh* trees is not as strong as it is for the clades in the mtDNA tree, but this probably reflects a reduced phylogenetic signal due to lower diversity in these sequences. Finally, the clades are not merely geographically defined. For example, sequences from each of the three chimpanzee parasite clades include samples from all four *Pan troglodytes* subspecies from sample sites as far apart as Cote d’Ivoire and western Uganda [25,30]. In light of the evidence shown above, it is clearly not appropriate to use *P. reichenowi* as an ‘umbrella term’ covering multiple lineages of ape *Plasmodium* parasites [22].

Initially, we termed the six *Laverania* clades C1–C3 and G1–G3, depending on whether they comprised chimpanzee or gorilla samples, respectively [25]. Clade C1 includes the CDC isolate of *P. reichenowi*, and should retain that historical name. Sequences falling within clades C2 and C3 have previously been termed *P. gaboni* [21], and *P. billcollinsi* [13], respectively. However, sequences corresponding to another recently proposed species, *P. billbrayi* [13], fall within the C2 clade and do not seem to be sufficiently distinct from *P. gaboni* to warrant a separate species designation at this time. The three species from the gorilla have not yet been named. For G2 and G3, we propose *P. adleri* and *P. blacklocki*, respectively, in honor of Saul Adler (1895–1966) and Donald Blacklock (1879–1953), two of the pioneers of malaria research in apes. The third clade of gorilla parasites (G1) includes human *P. falciparum*. This clade appears to have originated in gorillas, with human *P. falciparum* parasites having arisen from a single cross-species transmission (see below) such that they may now be effectively isolated from their gorilla precursors. If so, then the human and gorilla parasites within the G1 clade also represent distinct species. In this case, we propose the name *P. praefalciparum* for the parasites infecting gorillas to emphasize their role as the precursor of human *P. falciparum*. This nomenclature is summarized in Figure 2.

## The origin of *P. falciparum*

Sequences from human strains of *P. falciparum* are closely related to sequences of parasites from western gorillas [24,25], but other closely related sequences have also been reported from chimpanzee [23] and bonobo samples [13]. Various authors have argued that each of these three ape species were the primordial hosts of this clade. In addition, it has most recently been suggested that all of these ape infections with *P. falciparum-*like parasites are a very recent phenomenon ‘occurring only as a result of forest destruction that has brought human and ape populations into greater proximity, thus facilitating the transmission of human *P. falciparum* to gorillas’ [31]. However, existing lines of evidence clearly implicate gorillas as the original source of these parasites.

The *P. falciparum*-like sequences form the clade previously termed G1 [25]. G1 sequences were obtained from four bonobos who had been housed at a sanctuary on the outskirts of a major city (Kinshasa, Democratic Republic of the Congo), and from two chimpanzees who were kept as pets before sampling [13,23]. However, no G1 sequences were found in >1,800 samples from wild chimpanzees and no *Laverania* sequences of any kind were detected in samples from wild bonobos [25]. This strongly suggests that the six sanctuary apes acquired human-derived *Plasmodium* infections while in captivity. This hypothesis seems to have been confirmed by the observations that these G1 sequences from bonobos and chimpanzees fall within the radiation of sequences from human strains [25], and that the bonobo strains carried drug resistance mutations found in human *P. falciparum* [13].

In striking contrast, the gorilla-derived G1 sequences came from numerous wild-living individuals sampled at 11 sites across the range of the western gorilla in Cameroon, the Central African Republic, and the Republic of Congo [25]. These G1 sequences from gorillas exhibit high levels of mtDNA diversity, similar to those seen in other ape *Laverania* species, such as *P. reichenowi* in chimpanzees. Conversely, human-derived G1 sequences (i.e. human *P. falciparum*) are much less diverse, and in phylogenetic trees form a single well-supported clade within the radiation of gorilla-derived G1 sequences [25]. These observations would be difficult to explain if humans were the original hosts of the G1 clade, and especially if, as suggested [31], transmission to gorillas had occurred only very recently. Thus, all the available evidence indicates that western gorillas served as the source of human *P. falciparum*, and not the other way around. Also, there is not a single confirmed case of human *P. falciparum* infection in wild-living gorillas*.* Although one study reported two gorilla-derived G1 sequences that were identical to human *P. falciparum* [24], the amplified fragment was too short to differentiate human from gorilla G1 parasites [25].

## Host species specificity

The apparent host specificity of the *Laverania* species could be due to incompatibility at the parasite–host and/or the vector–host interface. It has been suggested that *P. reichenowi* and *P. falciparum* are specific to their respective chimpanzee and human hosts because of incompatibility at the erythrocyte entry stage. Invasion of human erythrocytes by *P. falciparum* merozoites relies heavily (although not exclusively) upon the interaction between the parasite ligand EBA-175 and its erythrocyte sialoglycoprotein receptor, glycophorin A (GPA) [32]. Sialic acid residues are present in one of two forms, the precursor form, *N*-aceytlneuraminic acid (Neu5Ac), or its modified product, *N*-glycolylneuraminic acid (Neu5Gc); the former is converted to the latter by an enzyme encoded by the CMAH gene [33,34]. Ape sialoglycoproteins contain predominantly Neu5Gc but the human CMAH gene contains an inactivating insertion, so human sialoglycoproteins contain only Neu5Ac residues [35]. Experimental studies have suggested that this Neu5Ac/Neu5Gc difference renders the major *P. falciparum* ligand EBA-175 unable to bind to chimpanzee GPA, whereas the *P. reichenowi* homolog of EBA-175 cannot bind to human GPA [36]. This sialic acid hypothesis cannot explain the host preferences of the various ape *Laverania* species because chimpanzees and gorillas express similar repertoires of sialic acids [35]. However, adaptation to the human sialic acid repertoire could have played a part in the isolation of human *P. falciparum* from its gorilla-infecting precursor, just as it was originally proposed to have had a role in the speciation of *P. falciparum* from *P. reichenowi* [36,37]. With our new understanding of the diversity of *Laverania* parasites, assessing the receptor-binding specificity of all parasites will be an important test to validate the sialic acid hypothesis.

## Natural history of ape *Plasmodium* infections

Non-invasive studies of *Plasmodium* infections have also provided the first estimates of *Laverania* prevalence rates in wild apes [25,30]. The results were striking: *Laverania* parasites were not only widely distributed but also highly prevalent in chimpanzees and western gorillas (Figure 1). Moreover, the reported prevalence rates represent minimum estimates because the extent to which infected apes shed *Plasmodium* DNA into their feces is not known. What do these prevalence rates tell us about the pathogenesis of *Laverania* parasites in chimpanzees and gorillas? Given the extremely high infection rates in some communities, it is probably safe to assume that severe malaria is not a frequent outcome of ape *Laverania* infections. No pathological consequences were noted in early studies of naturally infected apes [1], although the small numbers of cases examined and the frequent occurrence of co-infecting pathogens precluded definitive conclusions. Interestingly, *Laverania* infection rates in apes appear to be similar to *P. falciparum* prevalence rates in humans in hyperendemic transmission zones. In these regions, adults are largely immune to the clinical manifestations of malaria, but frequently maintain high-level *P. falciparum* infections in their blood [38]. It therefore seems probable that apes develop clinical immunity to *Laverania* parasites in much the same way that humans do. Thus, if *Laverania* parasites cause mortality in apes, it would be expected to occur in infants because in humans most *P. falciparum* mortality occurs in children under the age of 5 years. The other potential complication of ape *Laverania* infections may be in pregnant females because *P. falciparum* can cause placental complications in infected women. Screening *Laverania* parasites for genes associated with pregnancy complications, such as the cytoadherence ligand var2CSA [39], may help to answer this question.

## Non-*Laverania* infections in wild apes

Recent studies have not only revealed a much greater diversity of *P. falciparum*-related ape parasites, but also uncovered non-*Laverania* species in these same populations. Screening samples of feces or blood from apes with consensus primers generally leads to the amplification of *Laverania* sequences, suggesting that these parasites are present at high titers, just as *P. falciparum* is usually the predominant parasite in human mixed-species infections [40]. However, parasites related to *P. ovale*, *P. vivax* and *P. malariae* have now also been found in samples from apes [30,41,42]. The presence of *P. vivax*-related parasites in African apes is of particular relevance because the origins of human *P. vivax* are controversial. Several studies have suggested an origin in South-East Asia because the closest known relatives of *P. vivax* infect primates in this region [43,44]. However, this seems difficult to reconcile with the near fixation of the Duffy negative allele in human populations in central Africa. The Duffy antigen is a chemokine receptor required by *P. vivax* merozoites to enter red blood cells. The very high frequency of the negative genotype is suggestive of a prolonged exposure to *P. vivax* in this region [45]. The new data raise the possibility that humans in west central Africa may have been exposed to *P. vivax*-like parasites from apes over an extended period of time.

The discovery of *P. ovale-* and *P. vivax*-related parasites in apes also resolves some of the confusion surrounding *P. schwetzi*, which has been variously described as *P. vivax-like* or *P. ovale*-like. It seems likely that, in at least some cases, researchers were viewing different species in different ape samples but calling them by the same name. This is not surprising given that the distinction between *P. ovale* and *P. vivax* in humans was disputed for many years [46] and it was not until the 1940s that the two were widely and consistently recognized as separate species [47]. Similarly, it is likely that ape parasites previously termed ‘*P. reichenowi*’ comprised representatives of different *Laverania* species. Obviously, a complete understanding of the diversity of these parasites (together with an appropriate nomenclature) will be critical to assess the risk of future zoonoses.

## Are humans susceptible to infection with ape *Plasmodium* parasites?

It is now apparent that *Plasmodium* parasites are widely distributed in wild-living great apes, with multiple *Laverania* and non-*Laverania* species present, often at the same location and in the same individual, with prevalence approaching 100% at some sites. This opens up the possibility of comparative studies of ape and human parasites that could provide new and important insights into the pathobiology of human malaria. However, one important question raised by the new findings is the same as that posed by Eduard Reichenow when he first observed these parasites: do they serve as a source for human infection?

Much is already known about the ability of ape *Plasmodium* parasites to infect humans. In the decades following their discovery, a series of transmission experiments involving apes and humans were carried out. While unthinkable today, these studies nevertheless generated very valuable information. In the case of *P. rhodaini*, the data were unequivocal, with numerous studies showing that this parasite transmitted readily between apes and humans in both directions [8,48–50]. Given the ease of these transfers, Rhodain even argued that *P. rhodaini* and *P. malariae* were the same species [48]. The data for *P. vivax* and *P. ovale* are also fairly clear (although experiments were carried out with ‘*P. schwetzi*’ which, as noted above, could have represented either one or both of these parasites). ‘*P. schwetzi*’ was transmitted from apes to humans on more than one occasion [11,51–53]. In at least one of these cases, *‘P. schwetzi*’ may have been a *P. vivax*-related parasite because transmissions were successful if the recipients were Caucasians but not if they were Africans, perhaps due to the Duffy negativity of the latter [53]. In other cases, the resulting blood infections in humans were distinctly *P. ovale*-like [11]. It is therefore possible that transmission experiments were undertaken with both parasites and that both *P. vivax* and *P. ovale* can be transmitted from apes to humans.

The potential of *Laverania* parasites to cross between different hosts is less clear. As depicted in Figure 2, current evidence indicates that pandemic human *P. falciparum* arose after a single human-to-gorilla transmission event. The question is whether such jumps have occurred more than once. This has certainly been observed for other ape-derived zoonotic infections, including simian immunodeficiency viruses from chimpanzees and gorillas, which gave rise to human immunodeficiency virus (HIV)-1. These ape viruses have jumped to humans on at least four independent occasions, with the current AIDS pandemic being the result of only one such transmission event [54]. The fact that several attempts to infect humans with ‘*P. reichenowi*’ failed [2] does not argue against ape-to-human *Laverania* transmissions. All recorded cross-species infection experiments involved chimpanzee parasites, but precisely which chimpanzee *Laverania* species were tested is not known. These experiments therefore show that some chimpanzee *Laverania* parasites may not transfer readily to humans, but this may not be true for all of them. Most importantly, there is no record of an attempted transfer of ‘*P. reichenowi*’ from gorillas to humans.

## What is the likelihood of ape-to-human parasite transmission?

Given that human *P. falciparum* appears to have originated in gorillas, and that *P. malariae-*, *P. ovale-* and *P. vivax*-like ape parasites have been experimentally transmitted to humans on several occasions, the question arises: “to what extent are humans in west and central Africa exposed to infection with ape parasites?” In this context, vector susceptibility and host feeding preferences are important variables. Many successful ape–human transmission experiments were carried out with *Plasmodium*-infected blood, but some were carried out using infected mosquitoes [53]. Thus, ape *Plasmodium* parasites can spread to humans via the bites of at least some vector species. Under field conditions, cross-species transmission would require an *Anopheles* mosquito to bite an infected ape and to then take a human blood meal. Studies have addressed the extent to which various *Anopheles* species are susceptible to ape *Plasmodium* parasites. Importantly, attempts to inoculate ‘*P. reichenowi*’ into *A. gambiae* (the major human vector) failed [10,14,55]. However, it is currently not known which species transmit *Plasmodium* between wild apes. Hence, the ability of these mosquitoes to act as bridging vectors (which would have a major impact on the likelihood of ape-to-human transmission) remains to be determined.

**Figure 3:**
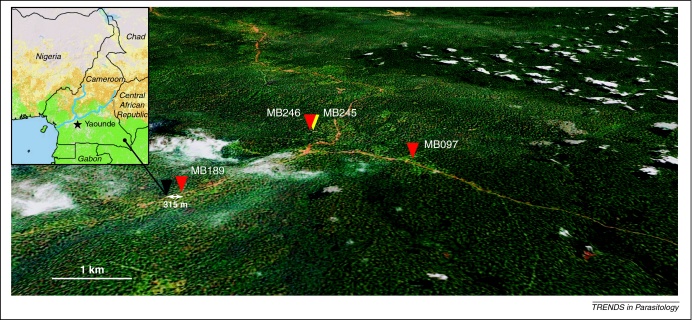
Proximity of *Plasmodium*-infected wild-living apes to human habitation. The location of chimpanzee fecal samples collected near a village (black triangle) in southeastern Cameroon (inset) is shown. *Plasmodium*-positive and -negative samples are depicted by red and yellow triangles, respectively. Samples are numbered with capital letters indicating the field site, with their location determined using global positioning system (GPS) coordinates. *Plasmodium*-positive samples were found within 315 m of human habitation and in close proximity to a nearby road (brown line). The map was generated using Google Maps Pro (the scale bar represents 1 km); white areas indicate cloud cover.

It will also be important to determine how many individuals live or work in close proximity to wild apes such that they would be exposed to *Anopheles* mosquitoes that have recently fed on apes. Dispersal of *Anopheles* in release–recapture experiments is 200–500 m [56] (although in some studies distances of over 1000 m have also been reported [57]). Humans living or working within 500 m of infected apes (including hunters, who routinely spend several days and nights in hunting camps in forests) are at greatest risk of exposure to ape *Plasmodium*-infected mosquitoes. Nevertheless, exposure is not limited to hunters because *Plasmodium*-positive fecal samples from apes have been collected within 500 m of human habitations (Figure 3). Depending on the dispersal characteristics of the respective vectors, exposure at much greater distances may also be possible [58].

The question thus arises why there is currently no evidence for zoonotic transmissions, particularly involving ape *Plasmodium* species where experimental infection has been documented. One answer is that most cases of malaria are not diagnosed but are treated presumptively based on symptoms. Moreover, ape *Plasmodium* parasites have not been exposed to drug-selection pressure. Hence, they would probably be sensitive to all anti-malarial agents, and would thus be eliminated by presumptive treatment even with chloroquine or sulfadoxine-pyrimethamine. Even in cases with positive blood smears, ape *Plasmodium* parasites would probably go unrecognized given their morphological similarity to human parasites. All original studies describing ‘*P. reichenowi*’ remarked on how similar this ape parasite was to human *P. falciparum*. It therefore seems likely that many (if not all) of the various *Laverania* species would be misidentified as *P. falciparum* by diagnostic microscopy. Similarly, ape-derived parasites related to *P. malariae*, *P. vivax* and *P. ovale* would probably look like their respective human counterparts and thus be diagnosed as such. Human infection with the macaque parasite *P. knowlesi* is an illuminating case in point. This zoonosis (which comprises several thousand cases every year) was initially misdiagnosed as *P. malariae* based on parasite morphology [59]. Even now, the diagnosis requires careful differential morphological examination combined with *Plasmodium* species-specific PCR [60]. Such approaches have not been used in regions of ape/human co-habitation in Africa.

## The way forward: an action plan

Numerous individuals in west and central Africa live and work in areas where they may encounter mosquitoes that have fed on *Plasmodium*-infected apes. Experiments carried out in the first half of the last century established that some ape *Plasmodium* parasites can be transmitted to humans. For others (including the gorilla parasite that gave rise to human *P. falciparum*), experimental data are lacking. Given the magnitude of the ape *Plasmodium* reservoir and the fact that a gorilla *Plasmodium* has crossed the species barrier to humans at least once, the question arises whether additional cross-species transfers of ape parasites have occurred or are occurring.

What needs to be done to address this question definitively? The distribution, prevalence and host associations of *Laverania* species have been determined [25]. Hence, it is important to screen wild apes for *P. vivax-*, *P. ovale-* and *P. malariae*-related parasites because it is for these species that the experimental evidence of cross-transmission is the strongest. Additional sequence information is also needed to provide a clearer picture of their phylogenetic relationships to the respective human infections, which will be informative as to whether ape-to-human and/or human-to-ape transfers have occurred. In the case of *P. vivax*-related parasites, this is of particular interest because human *P. vivax* is supposedly absent from west and central Africa [61]. In the case of *P. ovale*, which was recently proposed to comprise two separate species [26,62], this may help to illuminate the origins of these parasites. It is also likely, given the diversity of parasites identified so far, that additional ape *Plasmodium* species will be identified in African apes.

Secondly, humans living near wild-ape communities will need to be tested for zoonotic infections using molecular approaches capable of differentiating ape parasites. Mitochondrial polymorphisms unique to ape *Laverania* parasites may prove useful for this purpose [25]. Human *P. falciparum* infections in hyperendemic transmission areas frequently comprise mixtures of multiple genotypes. Detecting ape *Plasmodium* sequences in these mixtures may not be possible using standard PCR approaches, particularly if ape *Plasmodium* species are less fit than extant human *Plasmodium* parasites and therefore might be present at very low levels. ‘Next-generation’ sequencing such as 454 pyrosequencing, which generates tens of thousands of sequence reads from a single sample, could reveal such low-abundance infections. Characterizing the ‘microbiome’ of within-host *Plasmodium* diversity will also reveal new insights into the complexity and ecology of human mixed-parasite infections [63].

Finally, the mosquito species that transmit *Plasmodium* parasites among African apes will need to be identified in order to establish their potential to act as bridging vectors. Candidates include *A. moucheti* and *A. nili*, two known human malaria vectors found in and around forests across the area of west and central Africa that overlaps the ranges of the great apes [64]. However, current understanding of forest-dwelling *Anopheles* species is too limited to draw definitive conclusions. An active program that entails capturing *Anopheles* in close proximity of ape nesting sites and analyzing them for *Plasmodium* infections using molecular tools is clearly needed.

The approaches outlined above, together with biological studies of erythrocyte invasion and cytoadherence, promise to provide a comprehensive dataset to assess the risk of ape *Plasmodium* zoonoses. This information is of considerable public health importance not because such transmissions would be expected to contribute significantly to current malaria morbidity and mortality but because they would give an indication of the potential of ape malaria parasites to colonize humans in the future. This question of the risk and scale of potential zoonoses is particularly timely because plans for eradication of *P. falciparum* are beginning to be formalized [65]. If these programs are successful in reducing transmission within local human populations, they could open-up new niches for previously limited cross-species transfers to expand and potentially re-seed humans with new infections. Eduard Reichenow's question, first posed nearly a century ago, will finally be addressed, but with the benefit of a considerably advanced toolkit.

## Figures and Tables

**Figure I:**
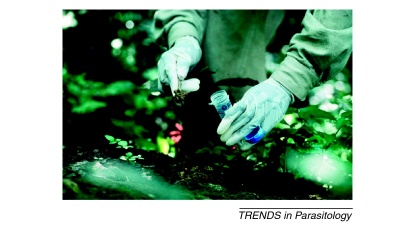
Non-invasive testing of ape *Plasmodium* infections. An ape fecal sample is collected at a remote forest site by an experienced tracker and preserved for shipment and storage in RNA*later* (Ambion).

## Box 1 Approaches to studying Plasmodium infection in apes

All African apes are highly endangered, thus precluding invasive studies of wild populations. Given this, options to study ape *Plasmodium* infections are limited. One strategy is to partner with ape sanctuaries and analyze specimens collected for diagnostic purposes as part of routine health measures. The advantage of this approach is access to blood samples, which may yield parasite isolates as well as *Plasmodium* sequences. The disadvantage is that sanctuary apes live in close proximity to humans and may thus be exposed to, and possibly infected by, human parasites. The other strategy is to screen ape fecal samples collected from the forest floor (Figure I) or, in the case of habituated communities, tissue samples from apes that are found dead, for *Plasmodium* infections [22,30]. The advantage of this approach is that much larger numbers of apes can be studied, and that all individuals are sampled within their natural habitat. The disadvantage is that parasite loads may be much lower than in blood samples, and that morphological characterization of parasites is not possible.
